# Brain activity during divided and selective attention to auditory and visual sentence comprehension tasks

**DOI:** 10.3389/fnhum.2015.00086

**Published:** 2015-02-19

**Authors:** Mona Moisala, Viljami Salmela, Emma Salo, Synnöve Carlson, Virve Vuontela, Oili Salonen, Kimmo Alho

**Affiliations:** ^1^Division of Cognitive Psychology and Neuropsychology, Institute of Behavioural Sciences, University of HelsinkiHelsinki, Finland; ^2^Department of Teacher Education, University of HelsinkiHelsinki, Finland; ^3^Advanced Magnetic Imaging Centre, Aalto NeuroImaging, Aalto UniversityEspoo, Finland; ^4^Brain Research Unit, Department of Neuroscience and Biomedical Engineering, Aalto University School of ScienceEspoo, Finland; ^5^Neuroscience Unit, Institute of Biomedicine/Physiology, University of HelsinkiHelsinki, Finland; ^6^Helsinki Medical Imaging Center, Helsinki University Central HospitalHelsinki, Finland; ^7^Helsinki Collegium for Advanced Studies, University of HelsinkiHelsinki, Finland; ^8^Swedish Collegium for Advanced StudyUppsala, Sweden

**Keywords:** dual-tasking, divided attention, selective attention, functional MRI, semantic processing

## Abstract

Using functional magnetic resonance imaging (fMRI), we measured brain activity of human participants while they performed a sentence congruence judgment task in either the visual or auditory modality separately, or in both modalities simultaneously. Significant performance decrements were observed when attention was divided between the two modalities compared with when one modality was selectively attended. Compared with selective attention (i.e., single tasking), divided attention (i.e., dual-tasking) did not recruit additional cortical regions, but resulted in increased activity in medial and lateral frontal regions which were also activated by the component tasks when performed separately. Areas involved in semantic language processing were revealed predominantly in the left lateral prefrontal cortex by contrasting incongruent with congruent sentences. These areas also showed significant activity increases during divided attention in relation to selective attention. In the sensory cortices, no crossmodal inhibition was observed during divided attention when compared with selective attention to one modality. Our results suggest that the observed performance decrements during dual-tasking are due to interference of the two tasks because they utilize the same part of the cortex. Moreover, semantic dual-tasking did not appear to recruit additional brain areas in comparison with single tasking, and no crossmodal inhibition was observed during intermodal divided attention.

## Introduction

Simultaneously performing several tasks is demanding and often leads to decrements in performance speed and accuracy (Pashler, 1994). These decrements may be due to a bottleneck in executive task-coordination systems recruited by multitasking (D’Esposito et al., 1995; Collette et al., 2005). Additional interference may be generated if the component tasks are presented in different sensory modalities and the corresponding sensory cortices have to compete for attentional resources (e.g., Näätänen, 1992). Competition may also occur beyond the sensory cortices in brain areas related to carrying out the component tasks in case these component tasks require similar (e.g., phonological or spatial) processing (Baddeley and Hitch, 1974). In the current study, we compared performance and brain activity in conditions requiring intermodal selective attention to one task with those demanding crossmodal division of attention between two simultaneous tasks requiring similar semantic processing. We asked (i) whether dividing attention recruits specialized executive task-coordinating systems; (ii) how attention modulates activity in the sensory cortices during bimodal linguistic stimulation; and (iii) how brain areas showing attention-related and task-specific activations react when two simultaneous tasks requiring similar processing are performed.

Previous research has suggested that multitasking recruits brain areas specialized in task coordination and managing interfering information from the component tasks (Corbetta et al., 1991; D’Esposito et al., 1995; Yoo et al., 2004; Stelzel et al., 2006). It has been suggested that dual-tasking involves task-coordinating abilities that are distinct from other executive functions such as shifting or inhibition (Miyake et al., 2000). Previous studies have highlighted the importance of frontal and parietal cortical areas as parts of a neural network involved in coordination of multiple parallel tasks. The involved frontal areas include the inferior frontal (Herath et al., 2001; Schubert and Szameitat, 2003; Stelzel et al., 2006) and middle frontal regions (Szameitat et al., 2002; Yoo et al., 2004) and the dorsolateral prefrontal cortex (Corbetta et al., 1991; D’Esposito et al., 1995; Johnson and Zatorre, 2006). The involved parietal areas, in turn, include the superior parietal lobule (Yoo et al., 2004) and intraparietal sulcus (Szameitat et al., 2002). The existence of specialized multitasking areas has been questioned, however, by studies failing to show multitasking-related activity in areas beyond those activated by the component tasks (Klingberg, 1998; Adcock et al., 2000; Bunge et al., 2000; Nijboer et al., 2014). These studies have shown that the performance of two concurrent tasks results only in a surplus of activation in the regions activated by the component tasks when performed separately, and no additional cortical regions are recruited. The former studies suggest that the main factor limiting performance during multitasking is the involvement of general coordinating or executive functions, whereas the latter studies suggest that limited task-specific resources are responsible for the observed interference during multitasking.

It has been repeatedly shown that when selective attention is directed to one modality, activity elevations in the sensory cortical areas processing attended inputs may be accompanied by diminished activity in the sensory cortical areas processing unattended inputs (Laurienti et al., 2002; Crottaz-Herbette et al., 2004; Shomstein and Yantis, 2004; Johnson and Zatorre, 2005; Mittag et al., 2013; Salo et al., 2013). It is less clear, however, how activity is modulated in the sensory cortices when attention is divided between two modalities. If there is a limited attentional resource allocated to the sensory cortices, sensory activity should decrease during intermodal divided attention when compared with selective attention to one modality. Indeed, there are studies showing such an effect during bimodal attention tasks (Loose et al., 2003; Johnson and Zatorre, 2006).

Many of the previous studies examining multitasking effects have used component tasks that do not necessarily rely on the same cortical areas, such as a semantic categorization task and a face recognition task (Adcock et al., 2000), or a spatial rotation and semantic judgment task (D’Esposito et al., 1995). It is therefore still unclear how task-related cortical activations are affected when several tasks competing for the use of those areas are performed simultaneously. In the current study, our participants performed two simultaneous sentence congruence judgment tasks. This type of task, when performed separately as a single task, has been shown to activate cortical areas related to semantic processing (e.g., Kiehl et al., 2002; Humphries et al., 2007). Functional magnetic resonance imaging (fMRI) studies using semantic congruence manipulations have consistently observed greater hemodynamic activity for incongruent than congruent sentences. The most commonly found areas to show this effect are the left superior temporal and left inferior frontal gyri, both when the sentences are presented as written text (Baumgaertner et al., 2002; Kuperberg et al., 2003; Service et al., 2007) and when they are presented as speech (Ni et al., 2000; Cardillo et al., 2004). These activations might be related to the N400 event-related potential (ERP) response elicited, for example, by an incongruent last word of sentence (e.g., “the pizza was too hot to *sing*”; Kutas and Hillyard, 1980).

In the present fMRI study, participants performed a sentence comprehension task involving spoken or written sentences, or both. The participants’ task was to rate the sentences as congruent or incongruent in only one modality at a time, or in both modalities simultaneously. This experimental setup allowed us to address three separate research questions related to multitasking. First, we investigated brain activity during simultaneous performance of two tasks in comparison with brain activity during the same tasks when performed separately. This allowed us to determine whether any additional cortical areas would be recruited during the divided attention condition. Second, the issue of crossmodal suppression of sensory cortices during selective attention to one modality was addressed. By performing a separate analysis in the auditory and visual cortices, we expected to see crossmodal suppression in the auditory cortex during selective attention to the visual modality, and vice versa. Moreover, in accordance with the hypothesis of limited resources, we expected to observe smaller attention-related activity in the visual and auditory cortices during division of attention between the two modalities than during intermodal selective attention to the written and spoken sentences, respectively. Third, we examined modulation of brain activity associated with linguistic processing when participants perform two simultaneous sentence comprehension tasks. This was accomplished by comparing activity elicited by incongruent sentences with activity elicited by congruent sentences during selective attention, thereby presumably isolating brain areas related specifically to semantic processing, and then examining activity modulations in these areas during divided attention. We hypothesized that as the number of tasks requiring semantic judgments is increased from one to two, activity in semantic processing areas increases. We expected to see that this increase would be non-additive due to limited processing capacity, leading to deficits in performing two simultaneous semantic judgments.

## Materials and methods

### Stimuli

#### Visual stimuli

Visual stimuli used in the experiment were written sentences and sentence-like nonsense text. They were projected onto a mirror mounted on the head coil and presented in the middle of the screen (font: Arial, size 14). The size of the sentences at the viewing distance of ~40 cm was ~1.4° vertically and ~24° horizontally.

##### Text

Written sentences were either semantically congruent or incongruent sentences in Finnish. The incongruent sentences were created by taking a subset of the congruent sentences (e.g., “*This morning I ate a bowl of cereal”*) and replacing the last word of the sentences with a semantically incongruent (but syntactically plausible) word (e.g., “*This morning I ate a bowl of shoes*”). Each participant saw a total of 192 congruent sentences and 144 incongruent sentences, because in the dual-task conditions more congruent sentences were needed (for details see Section Procedure).

##### Nonsense text

Sentence-like nonsense text was created by randomly selecting a subset of the congruent written sentences and replacing each vowel in those sentences with a different vowel. This procedure resulted in nonsensical sentences with word lengths and letter frequencies similar to the Finnish language. Forty eight different nonsense written sentences were used.

#### Auditory stimuli

Auditory stimuli used in the experiment consisted of speech, nonsense speech, and music. All auditory stimuli were presented binaurally through insert earphones (Sensimetrics model S14; Sensimetrics, Malden, MA, USA). All auditory stimuli were broadband stimuli high-pass filtered with a cut-off at 100 Hz and low-pass filtered with a cut-off at 7000 Hz. The intensity of auditory stimuli was scaled so that their total power in RMS units, the square root of the mean of the squared signal, was similar (0.1). The intensity of the sounds was individually set to a loud, but pleasant level, and was ~80 dB SPL as measured from the tip of the earphones. All adjustments to the auditory stimuli were made using Audacity ^1^ and Matlab (Mathworks Inc., Natick, MA, USA) softwares.

##### Speech

Spoken sentences were semantically congruent or incongruent Finnish sentences spoken by a female native Finnish speaker. The incongruent sentences were created in a similar way as the incongruent written sentences, that is, by replacing the last word in the congruent sentences. Each participant heard a total of 192 congruent sentences and 144 incongruent sentences, because in the dual-task conditions more congruent sentences were needed (see Section Procedure for details).

##### Nonsense speech

The nonsense speech stimuli consisted of recorded nonsensical sentences (see above) spoken by a female native Finnish speaker. Each participant heard a total of 112 nonsense speech sentences. The lengths of the sentences were adjusted so that each sentence had a duration of 2.5 s.

##### Music

2.5-s excerpts of instrumental music were obtained from a free-source online music website. The music excerpts represented various genres from hip-hop to classical music. Forty eight music clips were used.

#### Functional localizers

Functional localizers were used in order to accurately localize the auditory and visual sensory cortices of each participant. The auditory functional localizer was created by phase-scrambling spoken sentences by chopping the signal into short (10 ms) time-windows and shuffling the segments (Ellis, 2010). The visual functional localizer was a contrast-reversing checkerboard flickering at 8 Hz. The size of the checkerboard was similar to written sentences (~1.4° × ~24°), and it was centered at the middle of the screen. The auditory and visual localizers were presented simultaneously for 2.5 s, followed by a 1-s fixation cross (~1.4° × ~1.4°) at the center of screen.

### Participants

Participants were 18 healthy volunteering adults (9 females), all right handed and native Finnish speakers between 21 and 34 years of age (mean age 26 years) with normal hearing, normal or corrected-to-normal vision, and no history of psychiatric or neurological illnesses. An informed written consent was obtained from each participant before the experiment. The experimental protocol was approved by the Coordinating Ethics Committee of The Hospital District of Helsinki and Uusimaa, Finland.

### fMRI/MRI data acquisition

Functional brain imaging was carried out with 3 T MAGNETOM Skyra whole-body scanner (Siemens Healthcare, Erlangen, Germany) using a 20-channel head coil. The functional echo planar (EPI) images were acquired with an imaging area consisting of 43 contiguous oblique axial slices (TR 2500 ms, TE 32 ms, flip angle 75°, voxel matrix 64 × 64, field of view 20 cm, slice thickness 3.0 mm, in-plane resolution 3.1 mm × 3.1 mm × 3.0 mm). Image acquisition was performed at a constant rate, but was asynchronized with stimulus onsets. Four functional runs of 240 volumes were measured for each participant. A total of 960 functional volumes were obtained in one session (session duration approximately 37 min).

High-resolution anatomical images (voxel matrix 256 × 256, in-plane resolution 1 mm × 1 mm × 1 mm) were acquired from each participant between the third and fourth functional runs.

### Procedure

A total of ten experimental task blocks (each consisting of the nine experimental conditions with the divided attention condition repeated twice), one rest block, and one functional localizer block were included in each functional run. In the beginning of each block, instructions for the current task type were shown for 3.5 s. During the rest and localizer blocks, the participants were asked to look at the fixation cross. In subsequent task blocks, 12 sentences (visual or auditory) or sentence pairs (visual and auditory) were presented, each with a duration of 2.5 s. Each sentence was followed by a 1-s response window during which the participants were instructed to respond with an appropriate button press whether the attended sentence was congruent or not (or during the divided attention task whether both attended sentences were congruent or not) using their right index and middle finger, respectively. During the response window, a question mark (size 1.4° × 1.0°) was presented at the center of screen. The fixation cross preceded each written sentence for 500 ms on the left side of the screen where the first letter of the sentence subsequently appeared. When only speech stimuli were presented, the fixation cross was shown at the center of screen during the entire trial. At the end of each block, the participant was shown the percentage of correct responses in that block. The score was shown for 2 s, and followed by 4 s of rest before the next block.

A total of nine different experimental conditions were used. In the single-task conditions, the participants were instructed to attend to the sentences in just one modality (auditory or visual). There was either no stimuli presented in the other modality (the *unimodal condition*, two blocks), or distractor stimuli were present in the other modality and the participants were instructed to ignore them (the *selective attention condition*, two blocks). Auditory distractors were spoken sentences (one block), music (one block) or nonsense speech (one block). The visual distractors were written sentences (one block), which the participants were instructed to ignore by holding a steady fixation on a fixation cross presented in the middle of the screen. Two additional visual distractor conditions were included in order to control for eye movements: a moving fixation cross (one block) and the participants were instructed to follow it while attending to speech; nonsense written sentences (one block) and the participants were instructed to scan through the nonsense text while attending to speech. These two control conditions did not differ from the condition including written sentences as distractors and were therefore discarded from further analyses. In the *divided attention condition* (two blocks), the participants were presented with simultaneous spoken and written sentences and instructed to attend to both modalities, and asked to decide whether or not both sentences were congruent (both sentences were never incongruent).

There were four functional runs, 12 blocks in each run, and 12 trials (i.e., sentences, sentence pairs, or functional localizers) in each block. Each run included one block of each task type, except the divided attention task, which was repeated twice. This was done in order to ensure an equal amount of trials where the incongruent sentence was in the visual/auditory modality between the divided attention and the unimodal and selective attention condition blocks, since in the divided attention condition only half of the incongruent trials had an incongruent sentence in the visual/auditory modality. This resulted in a total of 96 trials for the divided attention task (4 × 2 × 12), and 48 trials for all the other task types (4 × 1 × 12). The order of tasks within the run was random, except that the rest block was always in the middle of the run between the 6th and 7th task block. All stimuli (sentences and distractors) were presented in randomized order. The sentences were randomized in the following way. First, the sentences were divided randomly into 4 sets (1 per run) that were identical for all participants. Then the order of sentences within a set was randomized, and the presentation order of these 4 sets was randomized and counterbalanced across participants. Each sentence was presented only once to each participant. The congruent and incongruent versions of the same sentence were never presented within the same run.

### fMRI data analysis

Image preprocessing and statistical analysis was performed using Statistical Parametric Mapping (SPM8) analysis package (Wellcome Department of Cognitive Neurology, London, UK; Friston et al., 1994a) as implemented in Matlab. In order to allow for initial stabilization of the fMRI signal, the first four dummy volumes were excluded from analysis. In pre-processing, the slice timing was corrected, data were motion corrected, high-pass filtered (cut-off at 1/128 Hz), and spatially smoothed with a 6 mm Gaussian kernel. The EPI images were intra-individually realigned to the middle image in each time series and un-warping was performed to correct for the interaction of susceptibility artifacts and head movements.

For the first-level statistical analysis, the general linear model was set up including a regressor for incongruent and congruent sentences in each of the 9 different and analyzed experimental conditions, resulting in 18 regressors. Separate regressors for the responses of the participants and for instructions (2.5-s periods between the blocks and a 6-s period at the beginning of each run) were also included. 6 movement parameters were added to the model as nuisance regressors. The regressors were convoluted with the canonical hemodynamic response function.

In the second-level analysis, the anatomical images were normalized to a canonical T1 template (MNI standard space) provided by SPM8 and then used as a template to normalize the contrast images for each participant (tri-linear interpolation, 3 mm × 3 mm × 3 mm using 16 nonlinear iterations). Statistical parametric maps of individual contrasts between task types and between tasks and rest were then averaged across participants. A voxel-wise height *t*-value threshold and a cluster size threshold were set depending on the contrast type (the specific values are stated below each contrast image). The statistical images were cluster corrected at *p* < 0.005 (Friston et al., 1994b). Anatomical regions corresponding to the activity foci were identified using the xjView toolbox for SPM.^2^

### Region of interest analysis

To study activity modulations in areas specifically related to dual-tasking, the divided attention condition was contrasted separately with the selective attention to text condition and the selective attention to speech condition. *Dual-tasking regions of interest* (ROIs) were then drawn manually using Freesurfer software to cover areas showing overlap between these two contrasts. Further statistical analyses were conducted using repeated-measures analyses of variance (ANOVAs) for voxels within these ROIs. Activity modulations between task conditions were compared by conducting an ANOVA with the factor Condition (9 levels) for each ROI separately and for data averaged across the ROIs. To compare activity modulations between the dual-tasking ROIs in the different task conditions, a 5 (Dual-tasking ROI) × 9 (Condition) ANOVA was conducted. Laterality effects in the dual-tasking ROIs were studied using a 2 (Hemisphere of the dual-tasking ROI) × 9 (Condition) ANOVA. To study the effects of attention in the unimodal, selective attention and divided attention conditions, a 5 (Dual-tasking ROI) × 3 (Task type) ANOVA was carried out. Finally, the effect of attended modality (irrespective of task type) was examined using a 5 (Dual-tasking ROI) × 3 (Attended modality) ANOVA. Note that the nine task conditions used in the ANOVAs also include the three conditions which were used to select dual-tasking ROIs.

ROI analyses were also conducted to examine activity modulations in the sensory cortices. To this end, voxels activated by the functional localizer (family-wise error corrected *p* < 0.05) were used as visual- and auditory-cortex ROIs of each individual participant. The mean percentage of blood-oxygen-level dependent (BOLD) signal change within the ROIs was calculated per voxel and normalized by dividing it by the overall average BOLD signal amplitude within a participant, and then averaged within each contrast of interest. To address the issue of possible crossmodal inhibition of the sensory cortices during selective attention, a 2 (Sensory cortex) × 2 (Attended modality) ANOVA was carried out for selective attention condition. To study dual-tasking effects, the divided attention condition was compared with the unimodal and selective attention conditions using an ANOVA with a 5 (Condition) × 2 (Hemisphere) × 2 (Sensory cortex) ANOVA.

ROI analysis was also used to study modulations of activity during divided attention in areas involved in semantic processing of sentences. In this analysis, contrasts between incongruent and congruent sentences in the second-level analysis were used to map areas of enhanced activity separately for written and spoken sentences. The incongruence contrast for speech sentences was created by summing together separately for attended incongruent and congruent sentences all the conditions where attention was directed to speech sentences (attention to speech in the unimodal condition, selective attention to speech with a text distractor, and the two additional visual distractor conditions) and all the conditions where attention was directed to written sentences (attention to text in the unimodal condition, selective attention to text presented together with speech, music or nonspeech distractors), and then contrasting the incongruent vs. congruent sentences within each modality. *Semantic* ROIs were drawn manually using Freesurfer software so that they covered areas showing overlap between the incongruence contrasts for spoken and written sentences. The mean percentage of BOLD signal change within each semantic ROI was calculated and normalized across the nine experimental conditions, and then averaged for each contrast of interest. To study the effects of the different task types on activity in the semantic ROIs, a 2 (Hemisphere of the ROI) × 3 (Task type) × 2 (Semantic congruence) ANOVA was carried out. Note that the three tasks types for an ANOVA were created by averaging the nine conditions used to select semantic ROIs.

### Analysis of behavioral data

The total percentage of correct responses per task type was calculated. The difference in the number of correct responses between task types was analyzed using a repeated-measures ANOVA with three Task levels (unimodal conditions vs. selective attention conditions vs. divided attention condition), where the two unimodal conditions where averaged together, and the six selective attention conditions were averaged together. An ANOVA was conducted on the three selective attention to text conditions (attention to text with a speech, nonsense speech or music distractor) in order to determine the effect of Auditory distractor type, and a similar ANOVA was conducted for the three selective attention to speech conditions (attention to speech with a text, nonsense text or moving fixation cross distractor) to study the effects of Visual distractor type. The effect of Attended modality was analyzed using an ANOVA with three levels (conditions where attention was targeted to written sentences vs. speech sentences vs. both written and speech sentences). The effect of the modality of incongruent sentences during the divided attention condition was analyzed using a paired sample *t*-test.

For all conducted ANOVAs the Greenhouse-Geisser *p*-value was used (as indicated by the correction value *ε*) if the Mauchly’s test of sphericity showed a significant result for a variable with more than two levels. However, original degrees of freedom will be reported with the *F*-value even in these cases. A 95% confidence interval was used in all ANOVAs. When an ANOVA yielded a significant result, Bonferroni *post hoc* tests were conducted. IBM SPSS Statistics 21 for Windows (IBM SPSS, Armonk, NY, USA) was used for statistical analyses.

## Results

### Behavioral results

**Figure 1:**
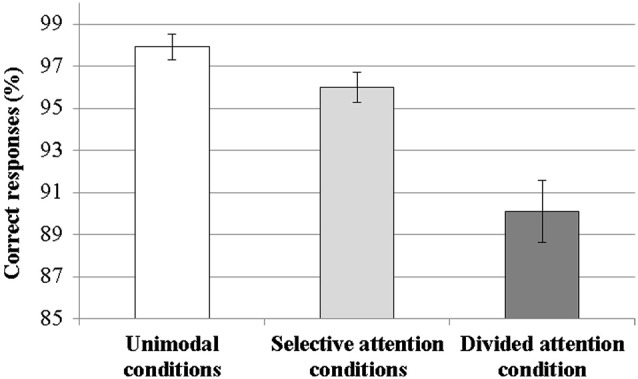
**Percentages of correct responses in the unimodal conditions (data combined across auditory and visual conditions), selective attention conditions (data combined across 6 conditions: selective attention to speech with a text, nonsense text or moving fixation cross distractor and selective attention to text with a speech, nonsense speech or music distractor) and divided attention condition**. Error bars indicate SEMs. Percentages of correct responses in the three condition types differed significantly from each other (in all cases, *p* < 0.05).

The mean percentage of correct responses (± standard error of the mean, SEM) was 97.6% ± 0.6% for the unimodal conditions, 95.3% ± 0.95% for the selective attention conditions, and 90.2% ± 1.6% for divided attention condition (Figure 1). The ANOVA with three Task levels showed a main effect of Task type (*F*_(2,32)_ = 23.69, *p* < 0.001) and subsequent *post hoc* tests revealed that the percentage of correct responses was significantly lower during divided attention than during attention in the unimodal condition (*p* < 0.001) or intermodal selective attention (*p* < 0.005) conditions, and significantly lower during selective attention than during attention in the unimodal condition (*p* < 0.05). The modality of the attended sentences did not affect the percentage of correct responses in single tasks (*p* = 0.24). The nature of the auditory distractor during selective attention to written sentences did not affect performance (*p* = 0.78). The ANOVA for auditory selective attention conditions showed a significant main effect of Visual distractor type (*F*_(2,32)_ = 6.31, *p* < 0.005, *ε* = 0.58) and *post hoc* tests indicated that significantly fewer correct responses were given when the visual distractor was regular written text than when it was a moving fixation cross or nonsense text (in both cases, *p* < 0.05).

Further analyses were conducted for the divided attention condition to study possible task strategy biases. These analyses showed that the percentage of correctly identified incongruent sentences did not depend on whether the incongruent sentence was in the visual or auditory modality (*p* = 0.24). Half of the participants had slightly higher accuracy rates (max. 12.5%) when the incongruent sentence was visual, and the other half when the incongruent sentence was auditory. Two participants performed at about chance level (46%) for auditory incongruent sentences, but the remaining participants had high response accuracies (>70%, mean 89%) for incongruent sentences in both modalities.

### Brain activity during divided vs. non-divided attention

**Figure 2:**
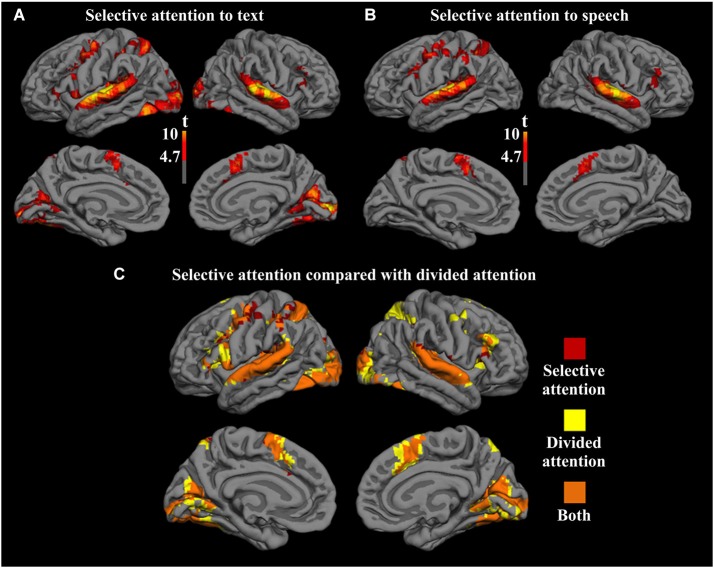
**Significant activity enhancements in relation to the rest blocks in the conditions (A) selective attention to text with a speech distractor and (B) selective attention to speech with a text distractor. (C)** A combination of these two contrasts is overlaid with the contrast showing activity enhancements during divided attention compared to rest. Areas showing significant activation enhancements only in the selective attention conditions are denoted with red and areas showing activation enhancements only in the divided attention condition are denoted with yellow. Areas showing overlap between these enhancements are denoted with orange. Voxel-wise height threshold *t* = 4.7, cluster size > 250, cluster-corrected *p* < 0.001.

Cortical networks recruited by selective attention to text with a speech distractor and selective attention to speech with a text distractor are shown in Figure 2. Activity during the selective attention tasks was compared with activity in the rest blocks. For the selective attention to text condition (Figure 2A), activity enhancement was seen bilaterally in the visual and auditory sensory cortices (BA 17/18/19, BA 41/42/22), and in the medial supplementary motor area (SMA; BA 6), precentral gyrus (BA 4/6), and inferior and middle frontal gyri (IFG and MFG; BA 44 and BA 46/9, respectively), and in the left superior and inferior parietal lobule (BA 7 and BA 40, respectively). A similar cortical network was activated by the selective attention to speech condition, with the exception of no significant activations in the visual sensory cortices (Figure 2B). Figure 2C shows comparisons between the areas recruited by the two selective attention conditions combined and the divided attention condition, demonstrating that these two networks largely overlap with each other. The activation map from the combined selective attention conditions compared with rest are denoted with red, and the activation map from the divided attention condition compared with rest is denoted with yellow. Areas showing overlap between these two contrasts are denoted with orange. This overlapping network includes bilaterally the visual and auditory cortices, medial SMA extending to more anterior regions of the medial superior frontal gyrus (BA 8/32), and the IFG and MFG, as well as the left precentral gyrus and superior and inferior parietal lobules.

**Figure 3:**
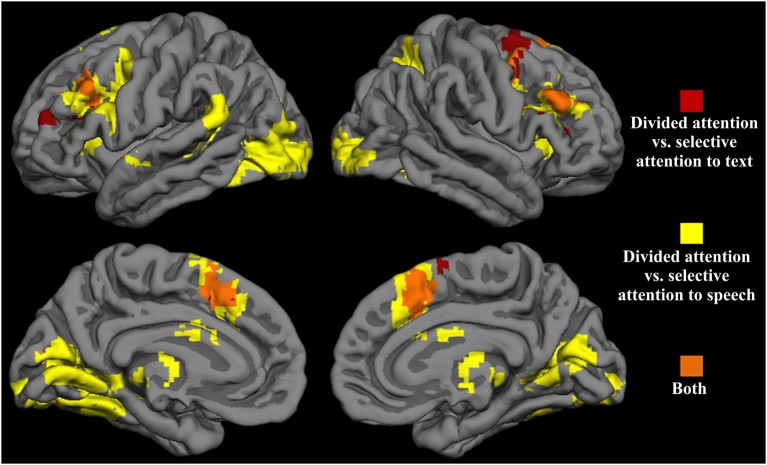
**Significant activity enhancements during divided attention in relation to selective attention to text with a speech distractor (red), selective attention to speech with a text distractor (yellow), and both (orange)**. Voxel-wise height threshold *t* = 2.5, cluster size > 250, cluster corrected *p* < 0.001.

Next, we contrasted the divided attention condition separately with the visual and auditory selective attention conditions with similar stimulation as during divided attention (i.e., selective attention to text with a speech distractor and selective attention to speech with a text distractor). The resulting contrast images were then overlaid on top of each other (Figure 3). Areas showing overlap between these two contrasts (orange areas in Figure 3) included clusters in the dorsolateral and medial portions of the frontal lobe. More specifically, clusters in the MFG (BA 9/6) and medial SMA (BA 6) showed greater activity bilaterally during divided attention than in either selective attention condition. Five dual-tasking ROIs were subsequently drawn to cover these regions showing overlap: the left and right anterior middle frontal gyrus (aMFG) ROIs, the left and right SMA ROIs, and the right posterior middle frontal gyrus (pMFG) ROI. Subsequent analyses were performed for voxels within these ROIs.

**Figure 4:**
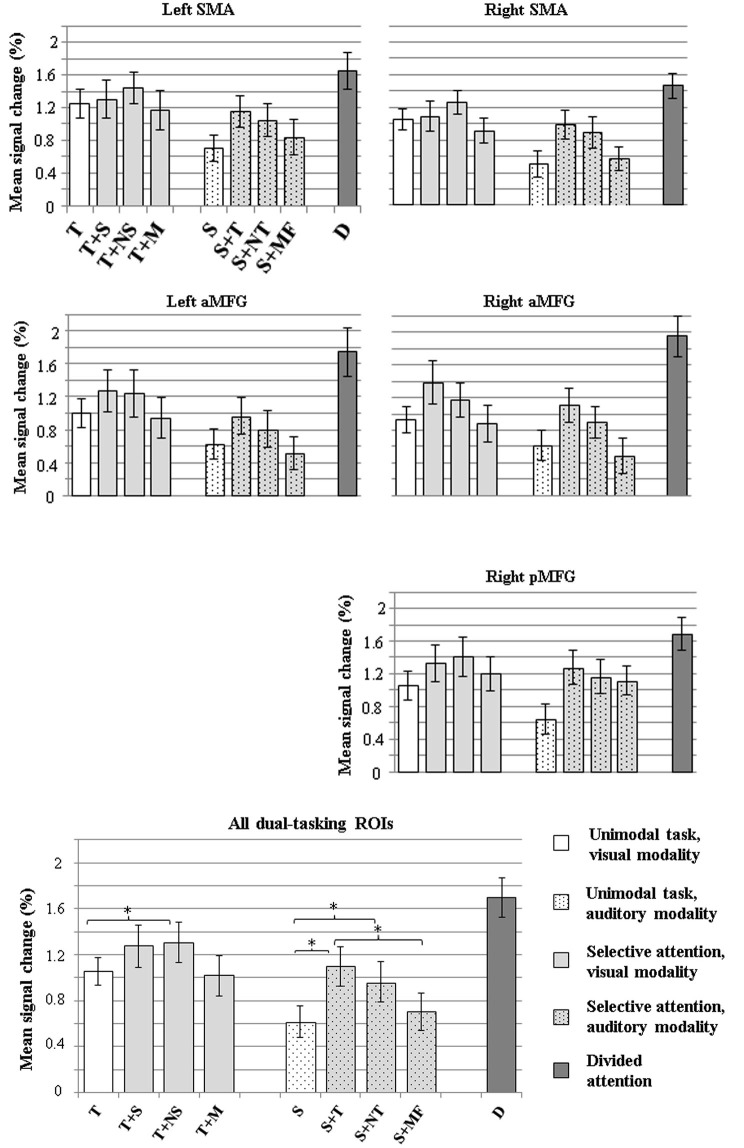
**Mean signal changes (%) compared with rest in the five dual-tasking ROIs during the nine experimental conditions**. Top: mean signal increases in the left and right SMA ROIs. Middle: mean signal increases in the left and right anterior MFG ROIs and the right posterior MFG ROI. Bottom: mean signal increases averaged across all five dual-tasking ROIs. The conditions in each graph are grouped based on the attended modality (left bar cluster: visual modality attended, middle bar cluster: auditory modality attended, rightmost bar: both modalities attended). Error bars indicate SEMs. Conditions differing significantly from each other are indicated with asterisks (*p < 0.05). Note that the nine conditions include the three conditions which were used to select dual-tasking ROIs. (T = attention to text in a unimodal condition, T+S = attention to text with a speech distractor, T+NS = attention to text with a nonsense speech distractor, T+M = attention to text with a music distractor, S = attention to speech in a unimodal condition, S+T = attention to speech with a text distractor, S+NT = attention to speech with a nonsense text distractor, S+MF = attention to speech with a moving fixation cross distractor, D = divided attention).

Figure 4 shows mean signal changes in the dual-tasking ROIs for each task condition. A significant main effect of Condition was observed for all five ROIs (for all ROIs, *p* < 0.001). There were no significant effects of Hemisphere for the MFG dual-tasking ROIs or SMA ROIs, or Condition × Hemisphere interactions. Since the five dual-tasking ROIs displayed a similar general pattern of activation for the different conditions (Figure 4, top and middle), and because no other main effects were observed, the data were averaged across the five dual-tasking ROIs in further analyses (Figure 4, bottom).

An ANOVA including Task type (unimodal vs. selective attention vs. divided attention) as the factor indicated a main effect of Task type (*F*_(2,34)_ = 47.72, *p* < 0.001). *Post hoc* tests revealed that the selective attention conditions resulted in significantly larger BOLD signal increases in the dual-tasking ROIs than the unimodal conditions (*p* < 0.05) and that divided attention was associated with greater signal increases than unimodal and selective attention conditions (in both cases, *p* < 0.001), which was expected since the dual-tasking ROIs were defined as areas showing greater activity during divided attention than selective attention. Another ANOVA with Attended modality (visual vs. auditory vs. both) as the factor indicated a significant main effect of Attended modality (*F*_(2,34)_ = 61.19, *p* < 0.001). Subsequent *post hoc* tests showed that BOLD signal increases in the dual-tasking ROIs were smallest in conditions where speech sentences were attended, followed by conditions where the text sentences were attended, and greatest when attention was divided between text and speech (in all cases, *p* < 0.001). When an ANOVA was conducted for data that were averaged across the dual-tasking ROIs (Figure 4, bottom), attending to text with a nonsense speech distractor caused greater BOLD signal increases than when no auditory distractor was present (*p* < 0.05). When speech was attended, both text and nonsense text distractors caused a greater signal increase than when no visual distractor was present (*p* < 0.05 in both). When the distractor was a moving fixation cross, signal increases did not differ from the condition with no visual distractor (*p* = 0.13), but were smaller than when a text distractor was present (*p* < 0.05).

### Attention effects on activity in the sensory cortices

When the activity in sensory cortices during the selective attention conditions (attention to text with a speech distractor and attention to speech with a text distractor) was examined, the interaction Sensory cortex (visual vs. auditory) × Attended modality (visual vs. auditory) was significant (*F*_(1,17)_ = 15.85, *p* < 0.001), that is, the visual cortex showed greater activity when attention was selectively directed to text than when it was directed to speech while the auditory cortex showed an opposite pattern.

**Figure 5:**
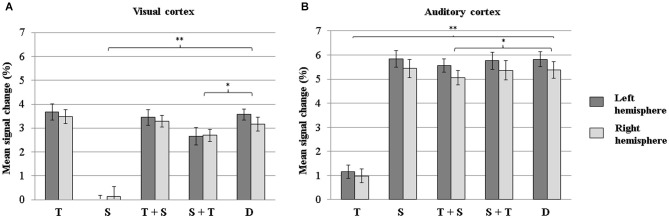
**Mean signal changes (%) in the visual (A) and auditory (B) cortices in the left and right hemispheres (dark gray and light gray bars, respectively) during attention to text in the unimodal condition, attention to speech in the unimodal condition, selective attention to text (with a speech distractor), selective attention to speech (with a text distractor), and divided attention**. Error bars indicate SEMs. Conditions differing significantly from the divided attention condition are indicated with asterisks (*p < 0.05; **p < 0.005). (T = attention to text in a unimodal condition, S = attention to speech in a unimodal condition, T+S = attention to text with a speech distractor, S+T = attention to speech with a text distractor, D = divided attention).

The results from the ANOVA including the factors Condition (attention to text in a unimodal condition vs. attention to speech in a unimodal condition vs. selective attention to text with a speech distractor vs. selective attention to speech with a text distractor vs. divided attention), Sensory cortex, and Hemisphere are illustrated in Figure 5. A significant main effect for Sensory cortex was observed (*F*_(1,17)_ = 43.53, *p* < 0.001), demonstrating that, overall, mean signal changes were greater in the auditory cortex than in the visual cortex. There was no significant main effect of Hemisphere (although there was some insignificant tendency for the left-hemisphere activity being higher than the right-hemisphere activity, *p* = 0.12). However, the main effect of Condition was significant (*F*_(4,68)_ = 63.04, *p* < 0.001, *ε* = 0.85). Subsequent pairwise comparisons revealed that the BOLD signal change was greatest during divided attention, followed by selective attention to text with a speech distractor, then by selective attention to speech with a text distractor, and lastly by attention to speech and attention to text in the unimodal conditions. Also, a significant interaction Condition × Sensory cortex was found (*F*_(4,68)_ = 190.12, *p* < 0.001, *ε* = 0.51). Pairwise comparisons revealed that in the visual cortex, the mean signal change during divided attention did not differ significantly from that during attention to the visual modality in the unimodal (*p* = 0.27) or selective attention condition (*p* = 0.98), but was significantly greater than that during auditory attention in the unimodal (*p* < 0.001) or selective attention condition (*p* < 0.005). Activity in the visual cortex during visual attention did not depend significantly on the presence of an auditory (speech) distractor (*p* = 0.27 for attention to text during selective attention vs. during the unimodal condition). In the auditory cortex, the mean signal change during divided attention did not differ significanly from that during attention to the auditory modality in the unimodal (*p* = 0.84) or selective attention condition (*p* = 0.83), but was signifcantly higher than that during visual attention in the unimodal (*p* < 0.001) or selective attention condition (*p* < 0.05). Activity in the auditory cortex during auditory attention did not depend significantly on the presence of a visual (text) distractor (*p* = 0.70 for attention to speech during selective attention vs. unimodal condition).

### Brain activity related to semantic processing

**Figure 6:**
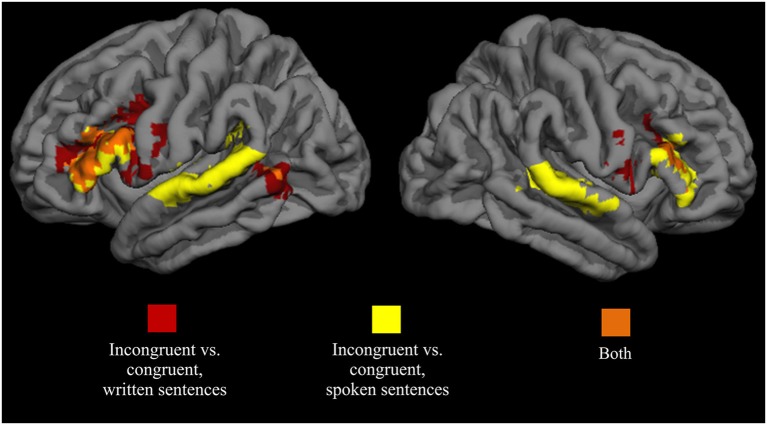
**Brain areas showing significant activity enhancements for attended incongruent written (red) and spoken (yellow) sentences (area overlaps shown in orange) in relation to respective congruent sentences**. Data combined across all single-task conditions for each modality. Voxel-wise height threshold *t* = 2.5, cluster size > 250, cluster-corrected *p* < 0.001.

As seen in Figure 6, analysis across the auditory single-task conditions showed that attended spoken incongruent sentences elicited a greater hemodynamic response than attended spoken congruent sentences bilaterally in the IFG (BA 44) extending to the MFG (BA 9/6), and in the superior temporal gyrus (BA 41/42/22). A similar comparison for attended written sentences in the visual single-task conditions showed activity enhancements for written incongruent sentences in relation to written congruent sentences bilaterally in the IFG (BA 44) extending to the MFG (BA 9/6), and in the posterior part of the left middle temporal gyrus (BA 21/37). When these two contrasts were overlaid (orange areas in Figure 6), two clusters corresponding roughly to the left and right IFG (BA 44) showed overlap between the two contrasts. In the left hemisphere, the overlap region covered both the pars opercularis and pars triangularis, and in the right hemisphere, the region was smaller and extended to the inferior frontal sulcus. Areas showing overlap were used as semantic ROIs and subsequent analyses were performed for voxels within these ROIs.

**Figure 7:**
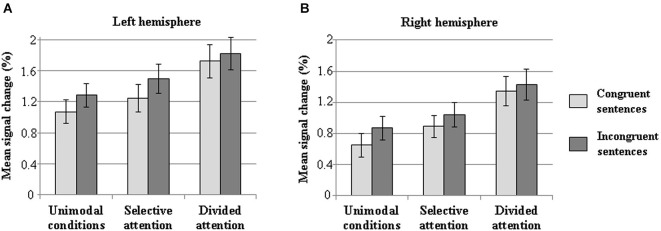
**Mean signal changes (%) in the semantic ROIs for attended incongruent and congruent sentences compared with rest during the unimodal (data combined across the unimodal auditory and visual conditions), selective attention (data combined across all auditory and visual selective attention conditions), and divided attention conditions in the left (A) and right (B) hemisphere**. Error bars indicate SEMs.

Mean signal changes in the semantic ROIs for congruent and incongruent sentences in the different task condition types are shown in Figure 7. As expected, the significant main effect of sentence congruence (*F*_(1,17)_ = 34.32, *p* < 0.001) confirmed that incongruent sentences caused greater increases in the BOLD signal than congruent sentences in both the left- and right-hemisphere semantic ROI. A main effect of Task type (*F*_(2,34)_ = 22.41, *p* < 0.001) revealed a greater increase in overall signal change during the divided attention condition than during the unimodal or selective attention conditions (*p* < 0.001 in both), and a greater increase during the selective attention conditions than unimodal conditions (*p* < 0.05) in the semantic ROI of each hemisphere. Also a main effect of Laterality was observed (*F*_(1,17)_ = 7.97, *p* < 0.05), demonstrating a greater signal change in the left-hemisphere semantic ROI than in the right-hemisphere semantic ROI. There were no significant interactions between the factors.

## Discussion

### Task performance

The behavioral results indicate that even though task performance was significantly worse during divided than during selective attention, the participants were still able to reach a high level of performance accuracy while attending to two stimuli simultaneously (even during divided attention mean response accuracy was over 90%).

### Divided attention vs. focused and selective attention

The difference between the selective attention and divided attention conditions was examined in order to determine whether any cortical activity was specifically related to dividing attention. Because in both conditions stimuli were presented in both modalities, the effect of sensory stimulation was controlled for in the contrast between these conditions. The results showed that divided attention recruited a very similar cortical network as the component tasks performed alone, since the activation maps showed a high degree of overlap.

When a direct comparison was made between the divided attention and the selective attention conditions, bilateral clusters both on the medial and dorsolateral frontal cortex showed significantly greater BOLD signal increases in the divided attention condition compared to the selective attention conditions. More specifically, these clusters were situated in the medial SMA and MFG of both hemispheres. The MFG has been implicated in memory rehearsal processes (Awh et al., 1996), rapid adaptation and coordination of actions required in dual-tasking (Szameitat et al., 2002), and detection of unexpected relevant stimuli (Corbetta and Shulman, 2002). The medial SMA, in turn, has been associated with performance monitoring, pre-response conflict, decision uncertainty, response errors, and processing of negative feedback (for a review, see Ridderinkhof et al., 2004). The need to inhibit a response to one sentence when it conflicts with the response to the other sentence, or the overall increase in difficulty in choosing the correct response in the divided attention condition might therefore explain the increase in SMA activity.

Areas showing higher activity during divided attention than during both selective attention to text and selective attention to speech were defined as dual-tasking ROIs. These ROIs were located in the medial SMA and MFG bilaterally. The smallest BOLD signal increases in these ROIs were seen during the unimodal conditions. The selective attention conditions activated these regions to a greater degree, with some activation differences that depended on the nature of the distractor stimuli. More specifically, nonsense speech as an auditory distractor and text and nonsense text as visual distractors caused greater activity increases than when no distractors were present. Since divided attention activated these ROIs the most, this might mean that these distractors were the most effective in drawing attention away from the actual task and creating a situation where attention was unintentionally divided between the attended and to-be-ignored modality.

Taking into account the high degree of overlap between the cortical networks activated by selective and divided attention, and the fact that dual-tasking ROIs showed a graded activation increase related to task difficulty (unimodal condition < selective attention < divided attention), our results suggest that at least semantic dual-tasking does not recruit new cortical areas, but places more demands on the brain regions already in use by the component tasks. This finding is in accordance with several previous studies showing that no additional neural regions are activated when interfering information needs to be coordinated (Klingberg, 1998; Adcock et al., 2000; Bunge et al., 2000; Nijboer et al., 2014), but rather that the component tasks compete for resources in a “global neuronal workspace” most likely located in frontoparietal regions (Hein et al., 2007). Some studies have reported opposite results, however, showing that frontal regions are recruited only during divided attention (Corbetta et al., 1991; D’Esposito et al., 1995; Herath et al., 2001; Szameitat et al., 2002; Schubert and Szameitat, 2003; Yoo et al., 2004; Johnson and Zatorre, 2006; Stelzel et al., 2006). These conflicting results may be explained more by the nature of the single tasks used in the individual studies than by the need to divide attention *per se*. Frontal recruitment may depend on the specific task demands of the single-tasks and vary from one task combination to the other. In our study, there are several possible explanations for the observed frontal recruitment during the component tasks. First, it could be related to inhibiting the processing of irrelevant information from the unattended modality. Frontal regions have been shown to be involved in gating sensory information according to task-specific demands (Miller and Cohen, 2001; Staines et al., 2002). Another possible explanation is catching of attention by stimuli in the unattended modality. It has been shown that a distributed network including frontal and parietal areas is activated when attention is involuntarily shifted to events in the sensory environment (Downar et al., 2000; Corbetta and Shulman, 2002; Salmi et al., 2009). The sentences in the unattended modality might therefore have caused an involuntary shift of attention to the unattended modality, resulting in frontal and parietal activity increases. Finally, our results might be explained by the difficulty of the component tasks used in the study. It could be argued that since our component tasks were complex sentence comprehension tasks, performing them required central executive functions to a great degree even in the absence of distracting stimuli or a need to divide attention between two modalities.

The frontoparietal cortical network observed in our selective attention and divided attention conditions bares a close resemblance to the multiple-demand (MD) network described by Duncan (2010). This general-purpose network includes cortex in and around the inferior frontal sulcus, the pre-SMA and the intraparietal sulcus, and it is activated by a variety of demanding cognitive tasks that require the formation of a series of subtasks. The tasks employed in our experiment can indeed be broken down into a succession of subtasks: internalizing the task instructions, evaluating the meaning of the presented sentence, choosing the correct response option, forming a motor response, reorienting to the next task instruction, etc. In the case of the present selective attention conditions, an additional subtask of inhibiting processing of the unattended stimulus is introduced. When two streams of stimuli have to be attended simultaneously, the amount of subtasks is even further increased even though the time given to complete these subtasks remains unchanged, adding to the demands placed on the MD network. It might therefore be that the observed BOLD signal increases in dorsolateral and medial frontal areas are a result of the task becoming more complex (i.e., involving more subtasks) and requiring quicker shifts from one subtask to the next, and not a result of a need to divide attention between two sensory streams.

The dorsolateral frontal activity increases during divided attention could also be explained by the recruitment of working memory when two tasks need to be performed simultaneously (Johnson et al., 2007). In our divided attention condition, the participants most likely had to maintain one sentence in a working memory buffer while making a congruence judgment concerning the other simultaneously presented sentence, whereas in the single-task condition no such demands were placed on working memory. In other words, the participants, at least some of them, may have adopted a rehearsal strategy during the divided attention task but not during the single-tasks. This could have led to the observed frontal activity increase, since the role of the dorsolateral prefrontal cortex in working memory (D’Esposito et al., 1995; Petrides, 2000) and more specifically in subvocal rehearsal (Awh et al., 1996) is well known. An experimental design specifically aimed at teasing apart the effects of increasing working memory load, divided attention, and overall task difficulty would be needed in order to determine the primary role of the dorsolateral prefrontal cortex in dual-tasking paradigms.

When interpreting our results with regards to dual tasking, it is important to note that the participants may not have been performing the divided attention task as the experimenters intended.

For example, the participants may have been attending to just one modality in the divided attention condition. Our behavioral results indicate that this is most likely not the case, however, because attending selectively to only one modality and performing at guess level for the other modality would have resulted in a response accuracy of 63–75%, a rate which our participants far surpassed. In addition, almost all participants performed at a high level of accuracy irrespective of the presentation modality of the incongruent sentence, demonstrating that there was no clear tendency to attend to just one modality. Another strategy used by our participants might have been to first attend to the written text and then switch to the unattended spoken sentence stored in a short-term memory (Norman, 1969), thus not really dual tasking but switching between the two tasks. The use of such a strategy might explain the increased parietal activity during dual tasking, as parietal regions have been shown to be involved in the voluntary shifting of attention between vision and audition (Shomstein and Yantis, 2004). On the other hand, it seems unlikely that such a strategy could have been used successfully in our experiment due to the fast pace of stimulus presentation. Participants had a total of 3.5 s per trial. The average length of the text sentences was 55 characters, which takes around 2.5 s to read at the average reading speed of Finnish text (Hahn et al., 2006). It is therefore unlikely that participants had had enough time to read out the spoken sentence from a short-term memory buffer after reading the text sentence, as subvocal rehearsal of auditory phonological material occurs in real time (Baddeley, 1992). In addition, if the text sentence was evaluated first, the participants would have likely detected incongruent written sentences significantly more accurately than incongruent spoken sentences, but according to our behavioral results this was not the case. As a final possible task strategy during dual tasking, our participants may have converted the written sentences they read into subvocalized speech rehearsed in the articulatory-phonological loop (Baddeley, 1992). If this indeed were the case, our divided attention task would not have been truly a bimodal one. However, even if this were the case, our main findings regarding brain activity associated with dual tasking would not be undermined, because our participants would still have been performing two tasks simultaneously albeit mainly in the same (auditory) modality.

### Attention effects in the sensory cortices

During bimodal stimulation when the participants were attending to just one sensory modality, the sensory cortical areas subserving the attended modality showed increased activity and the ignored sensory cortices showed a decrease in activity compared with when attention was directed to the other modality. This result is in accordance with previous studies showing a similar interaction between the attended modality and activity in the relevant sensory cortices (Shomstein and Yantis, 2004; Johnson and Zatorre, 2005, 2006; Salo et al., 2013).

The visual cortex was shown to be activated to the same extent during divided attention as during attention to visual stimuli in both the unimodal and selective attention conditions, and this activity was greater than when attention was directed to the auditory modality. An analogous pattern of results was observed for the auditory cortex. This result is in contrast to our initial hypothesis: Since several previous studies suggest that a common attentional resource is shared between the sensory modalities (Just et al., 2001; Loose et al., 2003; Johnson and Zatorre, 2006) we expected to see a decrease in sensory-cortex activations during divided attention in relation to auditory or visual selective attention. Our results also indicated that the addition of a distractor stimulus to the unattended modality did not affect activity in the sensory cortical areas subserving the attended modality, even though activity in the cortical areas processing the unattended stimuli increased significantly. If a common attentional resource were indeed shared among the different modalities, this would mean that no resources were allotted to the unattended modality. This would, however, make it difficult to account for the performance accuracy decrease seen in the selective attention condition compared with the unimodal condition. Therefore our results do not support the notion of a constrained total amount of attentional resources being spread out to all recruited sensory cortices.

### Activity related to semantic processing during divided attention

When only single-task conditions were examined, contrasting incongruent sentences with congruent sentences revealed an increase of activity in bilateral inferior frontal clusters for the written sentences, and in inferior frontal and temporal clusters for the spoken sentences. These foci of activity are well in line with the existing literature describing the role temporal and frontal areas (especially in the left hemisphere) in both semantic and syntactic language-related processing (Friederici et al., 2003; Hickok and Poeppel, 2004; for a review, see Vigneau et al., 2006). The increased activity in these areas in response to incongruent sentence endings is possibly due to the difficulty of integrating the unexpected last word to the preceding information, resulting in increased processing costs (Kutas and Hillyard, 1980). In electrophysiological studies, semantic integration was reflected as an increase in the amplitude of a specific ERP component, the N400 (Kutas and Hillyard, 1980; for reviews, see Kutas and Federmeier, 2000; Lau et al., 2008). The temporal activity clusters observed for the spoken sentences in our study is a likely candidate source for the N400 component (Humphries et al., 2006). The observed temporal activity could also be related to another ERP component, the phonological mismatch negativity (PMN; Connolly and Phillips, 1994), which is elicited when the initial phoneme of the last word in a sentence does not match the phoneme of the expected word (as was the case in our experiment). This component is elicited only when sentences are presented in the auditory modality, and it has been localized to the anterior superior temporal cortex predominantly in the left hemisphere (Kujala et al., 2004), and would therefore explain why we observed the temporal activity clusters only for the spoken sentences.

The IFG was activated bilaterally by both written and spoken incongruent sentences, this effect being stronger in the left than the right hemisphere. The important role of the IFG in processing the semantic content of linguistic stimuli has been demonstrated in previous studies (Baumgaertner et al., 2002; Kiehl et al., 2002). The IFG does not seem to contribute to the N400 component, however, as lesions to frontal areas including the IFG do not affect the N400 component (Friederici et al., 1999). Our results therefore add to the discrepancy between hemodynamic and electrophysiological studies describing the contribution of the IFG to semantic processing. Our study makes a valuable contribution to this debate, since we used both written and spoken sentence stimuli in the same study, and show that the IFG was activated for incongruent sentences irrespective of the presentation modality.

When two tasks that occupy a common part of the cortex are performed simultaneously, interference can occur at the level of these common regions (Roland and Zilles, 1998). In the case of our experiment, ROI analyses were conducted in the semantic ROIs (i.e., bilaterally in the IFG) during divided attention in order to study task interference more carefully. During divided attention, participants had to make two simultaneous or consecutive congruence judgments, presumably both relying on the same amodal semantic processing areas. When the overall activity in the semantic ROIs was examined, our results pointed to an increase in activity during divided attention when compared with the unimodal and selective attention conditions. This suggests that more demands were placed on semantic processing areas when two semantic tasks were performed in parallel, which possibly contributed to the observed performance decrements.

It is important to take into consideration the possibility that incongruent sentences elicited more IFG activity due to other cognitive functions than semantic processing. For example, it has been shown that the IFG is activated when prepotent responses are inhibited (Menon et al., 2001; Aron et al., 2004). Reading or listening to sentences where an anomaly occurs at the very end may create a situation where a response that the sentence is congruent is always chosen first, but then has to be inhibited and replaced by a new response when an anomaly is detected. This may explain the observed IFG activity enhancements. Yet another possible explanation relates to the observation that the IFG is involved with the detection of salient stimuli irrespective of task type (Hampshire et al., 2010). Sentences with semantic violations may represent such an unexpected and salient stimulus, thus involving the IFG.

## Conclusions

The participants of our study performed significantly more errors when they had to make two simultaneous sentence congruence judgments in separate modalities than when they performed just one such judgment in one modality. This dual-task interference could potentially be caused by mutual inhibition of the sensory cortices, or by the recruitment of additional cortical areas responsible for additional cognitive operations related to dual-tasking, or by interference of the two tasks because they utilize the same part of the cortex. Our results indicate that crossmodal inhibition of the sensory cortices is not responsible for the observed performance decrements, and that no dual-task-specific areas are recruited when attention is divided between two simultaneous semantic tasks involving parallel attention to speech and written text. Competition for resources in cortical areas used by both component tasks most likely contributes to dual-tasking interference.

## Conflict of interest statement

The authors declare that the research was conducted in the absence of any commercial or financial relationships that could be construed as a potential conflict of interest.

## Footnotes

^1^http://audacity.sourceforge.net

^2^http://www.alivelearn.net/xjview

## References

[B1] Adcock R. A. R. Constable T. Gore J. C. Goldman-Rakic P. S. (2000). Functional neuroanatomy of executive processes involved in dual-task performance. Proc. Natl. Acad. Sci. U S A 97, 3567–3572. 10.1073/pnas.97.7.3567 10725387 PMC16280

[B2] Aron A. R. Robbins T. W. Poldrack R. A. (2004). Inhibition and the right inferior frontal cortex. Trends Cogn. Sci. 8, 170–177. 10.1016/j.tics.2004.02.010 15050513

[B3] Awh E. Jonides J. Smith E. E. Schumacher E. H. Koeppe R. Katz S. (1996). Dissociation of storage and rehearsal in verbal working memory: evidence from positron emission tomography. Psychol. Sci. 7, 25–31 10.1111/j.1467-9280.1996.tb00662.x

[B4] Baddeley A. (1992). Working memory: the interface between memory and cognition. J. Cogn. Neurosci. 4, 281–288. 10.1162/jocn.1992.4.3.281 23964884

[B5] Baddeley A. D. Hitch G. (1974). “Working memory,” in The Psychology of Learning and Motivation: Advances in Research and Theory, ed Bower G. (New York, NY: Academic Press), 47–90.

[B6] Baumgaertner A. Weiller C. Büchel C. (2002). Event-related fMRI reveals cortical sites involved in contextual sentence integration. Neuroimage 16, 736–745. 10.1006/nimg.2002.1134 12169257

[B7] Bunge S. A. Klingberg T. Jacobsen R. B. Gabrieli J. D. (2000). A resource model of the neural basis of executive working memory. Proc. Natl. Acad. Sci. U S A 97, 3573–3578. 10.1073/pnas.97.7.3573 10725372 PMC16281

[B8] Cardillo E. R. Aydelott J. Matthews P. M. Devlin J. T. (2004). Left inferior prefrontal cortex activity reflects inhibitory rather than facilitatory priming. J. Cogn. Neurosci. 16, 1552–1561. 10.1162/0898929042568523 15601518 PMC2651466

[B9] Collette F. Olivier L. Van der Linden M. Laureys S. Delfiore G. Luxen A.. (2005). Involvement of both prefrontal and inferior parietal cortex in dual-task performance. Brain Res. Cogn. Brain Res. 24, 237–251. 10.1016/j.cogbrainres.2005.01.023 15993762

[B10] Connolly J. Phillips N. (1994). Event-related potential components reflect phonological and semantic processing of the terminal word of spoken sentences. J. Cogn. Neurosci. 6, 256–266. 10.1162/jocn.1994.6.3.256 23964975

[B11] Corbetta M. Miezin F. M. Dobmeyer S. Shulman G. L. Petersen S. E. (1991). Selective and divided attention during visual discriminations of shape, color and speed: functional anatomy by positron emission tomography. J. Neurosci. 11, 2383–2402. 1869921 10.1523/JNEUROSCI.11-08-02383.1991 PMC6575512

[B12] Corbetta M. Shulman G. L. (2002). Control of goal-directed and stimulus-driven attention in the brain. Nat. Rev. Neurosci. 3, 201–215. 10.1038/nrn755 11994752

[B13] Crottaz-Herbette S. Anagnoson R. T. Menon V. (2004). Modality effects in verbal working memory: differential prefrontal and parietal responses to auditory and visual stimuli. Neuroimage 21, 340–351. 10.1016/j.neuroimage.2003.09.019 14741672

[B14] D’Esposito M. Detre J. A. Alsop D. C. Shin R. K. Atlas S. Grossman M. (1995). The neural basis of the central executive system of working memory. Nature 378, 279–281. 10.1038/378279a0 7477346

[B15] Downar J. Crawley A. P. Mikulis D. J. Davis K. D. (2000). A multimodal cortical network for the detection of changes in the sensory environment. Nat. Neurosci. 3, 277–283. 10.1038/72991 10700261

[B16] Duncan J. (2010). The multiple-demand (MD) system of the primate brain: mental programs for intelligent behaviour. Trends Cogn. Sci. 14, 172–179. 10.1016/j.tics.2010.01.004 20171926

[B17] Ellis D. P. W. (2010). “Time-domain scrambling of audio signals in Matlab”. Available online at: http://www.ee.columbia.edu/~dpwe/resources/matlab/scramble/

[B18] Friederici A. D. Rüschemeyer S. A. Hahne A. Fiebach C. J. (2003). The role of left inferior frontal and superior temporal cortex in sentence comprehension: localizing syntactic and semantic processes. Cereb. Cortex 13, 170–177. 10.1093/cercor/13.2.170 12507948

[B19] Friederici A. D. von Cramon D. Y. Kotz S. A. (1999). Language related brain potentials in patients with cortical and subcortical left hemisphere lesions. Brain 122, 1033–1047. 10.1093/brain/122.6.1033 10356057

[B20] Friston K. J. Holmes A. P. Worsley K. J. Poline J. P. Frith C. D. Frackowiak R. S. (1994a). Statistical parametric maps in functional imaging: a general linear approach. Hum. Brain Mapp. 2, 189–210 10.1002/hbm.460020402

[B21] Friston K. J. Worsley K. J. Frackowiak R. S. J. Mazziotta J. C. Evans A. C. (1994b). Assessing the significance of focal activations using their spatial extent. Hum. Brain Mapp. 1, 210–220. 10.1002/hbm.460010306 24578041

[B22] Hahn G. A. Penka D. Gehrlich C. Messias A. Weismann M. Hyvärinen L.. (2006). New standardised texts for assessing reading performance in four European languages. Br. J. Ophthalmol. 90, 480–484. 10.1136/bjo.2005.087379 16547331 PMC1857021

[B23] Hampshire A. Chamberlain S. R. Monti M. M. Duncan J. Owen A. M. (2010). The role of the right inferior frontal gyrus: inhibition and attentional control. Neuroimage 50, 1313–1319. 10.1016/j.neuroimage.2009.12.109 20056157 PMC2845804

[B24] Hein G. Alink A. Kleinschmidt A. Müller N. G. (2007). Competing neural responses for auditory and visual decisions. PLoS One 2:e320. 10.1371/journal.pone.0000320 17389911 PMC1824707

[B25] Herath P. Klingberg T. Young J. Amunts K. Roland P. (2001). Neural correlates of dual task interference can be dissociated from those of divided attention: an fMRI study. Cereb. Cortex 11, 796–805. 10.1093/cercor/11.9.796 11532885

[B26] Hickok G. Poeppel D. (2004). Dorsal and ventral streams: a framework for understanding aspects of the functional anatomy of language. Cognition 92, 67–99. 10.1016/j.cognition.2003.10.011 15037127

[B27] Humphries C. Binder J. R. Medler D. A. Liebenthal E. (2006). Syntactic and semantic modulation of neural activity during auditory sentence comprehension. J. Cogn. Neurosci. 18, 665–679. 10.1162/jocn.2006.18.4.665 16768368 PMC1635792

[B28] Humphries C. Binder J. R. Medler D. A. Liebenthal E. (2007). Time course of semantic processes during sentence comprehension: an fMRI study. Neuroimage 36, 924–932. 10.1016/j.neuroimage.2007.03.059 17500009 PMC1941617

[B29] Johnson J. A. Strafella A. P. Zatorre R. J. (2007). The role of the dorsolateral prefrontal cortex in bimodal divided attention: two transcranial magnetic stimulation studies. J. Cogn. Neurosci. 19, 907–920. 10.1162/jocn.2007.19.6.907 17536962

[B30] Johnson J. A. Zatorre R. J. (2005). Attention to simultaneous unrelated auditory and visual events: behavioral and neural correlates. Cereb. Cortex 15, 1609–1620. 10.1093/cercor/bhi039 15716469

[B31] Johnson J. A. Zatorre R. J. (2006). Neural substrates for dividing and focusing attention between simultaneous auditory and visual events. Neuroimage 31, 1673–1681. 10.1016/j.neuroimage.2006.02.026 16616520

[B32] Just M. A. Carpenter P. A. Keller T. A. Emery L. Zajac H. Thulborn K. R. (2001). Interdependence of non-overlapping cortical systems in dual cognitive tasks. Neuroimage 14, 417–426. 10.1006/nimg.2001.0826 11467915

[B33] Kiehl K. A. Laurens K. R. Liddle P. F. (2002). Reading anomalous sentences: an event-related fMRI study of semantic processing. Neuroimage 17, 842–850. 10.1006/nimg.2002.1244 12377158

[B34] Klingberg T. (1998). Concurrent performance of two working memory tasks: potential mechanisms of interference. Cereb. Cortex 8, 593–601. 10.1093/cercor/8.7.593 9823480

[B35] Kujala A. Alho K. Service E. Ilmoniemi R. J. Connolly J. F. (2004). Activation in the anterior left auditory cortex associated with phonological analysis of speech input: localization of the phonological mismatch negativity response with MEG. Brain Res. Cogn. Brain Res. 21, 106–113. 10.1016/j.cogbrainres.2004.05.011 15325418

[B36] Kuperberg G. R. Holcomb P. J. Sitnikova T. Greve D. Dale A. M. Caplan D. (2003). Distinct patterns of neural modulation during the processing of conceptual and syntactic anomalies. J. Cogn. Neurosci. 15, 272–293. 10.1162/089892903321208204 12676064

[B37] Kutas M. Federmeier K. D. (2000). Electrophysiology reveals semantic memory use in language comprehension. Trends Cogn. Sci. 4, 463–470. 10.1016/s1364-6613(00)01560-6 11115760

[B38] Kutas M. Hillyard S. A. (1980). Reading senseless sentences: brain potentials reflect semantic incongruity. Science 207, 203–205. 10.1126/science.7350657 7350657

[B39] Lau E. F. Phillips C. Poeppel D. (2008). A cortical network for semantics: (de)constructing the N400. Nat. Rev. Neurosci. 9, 920–933. 10.1038/nrn2532 19020511

[B40] Laurienti P. J. Burdette J. H. Wallace M. T. Yen Y. F. Field A. S. Stein B. E. (2002). Deactivation of sensory-specific cortex by cross-modal stimuli. J. Cogn. Neurosci. 14, 420–429. 10.1162/089892902317361930 11970801

[B41] Loose R. Kaufmann C. Auer D. P. Lange K. W. (2003). Human prefrontal and sensory cortical activity during divided attention tasks. Hum. Brain Mapp. 18, 249–259. 10.1002/hbm.10082 12632463 PMC6871829

[B42] Menon V. Adleman N. E. White C. D. Glover G. H. Reiss A. L. (2001). Error-related brain activation during a Go/NoGo response inhibition task. Hum. Brain Mapp. 12, 131–143. 10.1002/1097-0193(200103)12:3<131::aid-hbm1010>3.0.co;2-c 11170305 PMC6872006

[B43] Miller E. K. Cohen J. D. (2001). An integrative theory of prefrontal cortex function. Annu. Rev. Neurosci. 24, 167–202. 10.1146/annurev.neuro.24.1.167 11283309

[B44] Mittag M. Inauri K. Huovilainen T. Leminen M. Salo E. Rinne T.. (2013). Attention effects on the processing of task-relevant and task-irrelevant speech sounds and letters. Front. Neurosci. 7:231. 10.3389/fnins.2013.00231 24348324 PMC3847663

[B45] Miyake A. Friedman N. P. Emerson M. J. Witzki A. H. Howerter A. Wager T. D. (2000). The unity and diversity of executive functions and their contributions to complex “frontal lobe” tasks: a latent variable analysis. Cogn. Psychol. 41, 49–100. 10.1006/cogp.1999.0734 10945922

[B46] Näätänen R. (1992). Attention and Brain Function. Hillsdale, NJ: Lawrence Erlbaum Associates.

[B48] Ni W. Constable R. Mencl W. Pugh K. Fulbright R. Shaywitz S.. (2000). An event-related neuroimaging study distinguishing form and content in sentence processing. J. Cogn. Neurosci. 12, 120–133. 10.1162/08989290051137648 10769310

[B49] Nijboer M. Borst J. van Rijn H. Taatgen N. (2014). Single-task fMRI overlap predicts concurrent multitasking interference. Neuroimage 100, 60–74. 10.1016/j.neuroimage.2014.05.082 24911376

[B50] Norman D. A. (1969). Memory while shadowing. Q. J. Exp. Psychol. 21, 85–93. 10.1080/14640746908400200 5777987

[B51] Pashler H. (1994). Dual-task interference in simple tasks: data and theory. Psychol. Bull. 116, 220–244. 10.1037//0033-2909.116.2.220 7972591

[B52] Petrides M. (2000). The role of the mid-dorsolateral prefrontal cortex in working memory. Exp. Brain Res. 133, 44–54. 10.1007/s002210000399 10933209

[B53] Ridderinkhof K. R. Ullsperger M. Crone E. A. Nieuwenhuis S. (2004). The role of the medial frontal cortex in cognitive control. Science 306, 443–447. 10.1126/science.1100301 15486290

[B54] Roland P. E. Zilles K. (1998). Structural divisions and functional fields in the human cerebral cortex. Brain Res. Brain Res. Rev. 26, 87–105. 10.1016/s0165-0173(97)00058-1 9651489

[B55] Salmi J. Rinne T. Koistinen S. Salonen O. Alho K. (2009). Brain networks of bottom-up triggered and top-down controlled shifting of auditory attention. Brain Res. 1286, 155–164. 10.1016/j.brainres.2009.06.083 19577551

[B56] Salo E. Rinne T. Salonen O. Alho K. (2013). Brain activity during auditory and visual phonological, spatial and simple discrimination tasks. Brain Res. 1496, 55–69. 10.1016/j.brainres.2012.12.013 23261663

[B57] Schubert T. Szameitat A. J. (2003). Functional neuroanatomy of interference in overlapping dual tasks: an fMRI study. Brain Res. Cogn. Brain Res. 17, 733–746. 10.1016/s0926-6410(03)00198-8 14561459

[B58] Service E. Helenius P. Maury S. Salmelin R. (2007). Localization of syntactic and semantic brain responses using magnetoencephalography. J. Cogn. Neurosci. 19, 1193–1205. 10.1162/jocn.2007.19.7.1193 17583994

[B59] Shomstein S. Yantis S. (2004). Control of attention shifts between vision and audition in human cortex. J. Neurosci. 24, 10702–10706. 10.1523/jneurosci.2939-04.2004 15564587 PMC6730120

[B60] Staines W. R. Graham S. J. Black S. E. McIlroy W. E. (2002). Task-relevant modulation of contralateral and ipsilateral primary somatosensory cortex and the role of a prefrontal-cortical sensory gating system. Neuroimage 15, 190–199. 10.1006/nimg.2001.0953 11771988

[B61] Stelzel C. Schumacher E. H. Schubert T. D’Esposito M. (2006). The neural effect of stimulus-response modality compatibility on dual-task performance: an fMRI study. Psychol. Res. 70, 514–525. 10.1007/s00426-005-0013-7 16175414

[B62] Szameitat A. J. Schubert T. Müller K. Von Cramon D. Y. (2002). Localization of executive functions in dual-task performance with fMRI. J. Cogn. Neurosci. 14, 1184–1199. 10.1162/089892902760807195 12495525

[B63] Vigneau M. Beaucousin V. Herve P. Y. Duffau H. Crivello F. Houde O.. (2006). Meta-analyzing left hemisphere language areas: phonology, semantics and sentence processing. Neuroimage 30, 1414–1432. 10.1016/j.neuroimage.2005.11.002 16413796

[B65] Yoo S. S. Paralkar G. Panych L. P. (2004). Neural substrates associated with the concurrent performance of dual working memory tasks. Int. J. Neurosci. 114, 613–631. 10.1080/00207450490430561 15204056

